# Forecasting future prevalence of type 2 diabetes mellitus in Syria

**DOI:** 10.1186/1471-2458-13-507

**Published:** 2013-05-25

**Authors:** Radwan Al Ali, Fawaz Mzayek, Samer Rastam, Fouad M Fouad, Martin O’Flaherty, Simon Capewell, Wasim Maziak

**Affiliations:** 1 Syrian Center for Tobacco Studies, Aleppo, Syria; 2 School of Medicine, University of Aleppo, Aleppo, Syria; 3 Division of Epidemiology and Biostatistics, School of Public Health, University of Memphis, Memphis, TN, USA; 4 Division of Public Health, University of Liverpool, Liverpool, United Kingdom; 5 Robert Stempel College of Public Health and Social Work, Florida International University, Miami, FL, USA

## Abstract

**Background:**

Type 2 diabetes mellitus (T2DM) is increasingly becoming a major public health problem worldwide. Estimating the future burden of diabetes is instrumental to guide the public health response to the epidemic. This study aims to project the prevalence of T2DM among adults in Syria over the period 2003–2022 by applying a modelling approach to the country’s own data.

**Methods:**

Future prevalence of T2DM in Syria was estimated among adults aged 25 years and older for the period 2003–2022 using the IMPACT Diabetes Model (a discrete-state Markov model).

**Results:**

According to our model, the prevalence of T2DM in Syria is projected to double in the period between 2003 and 2022 (from 10% to 21%). The projected increase in T2DM prevalence is higher in men (148%) than in women (93%). The increase in prevalence of T2DM is expected to be most marked in people younger than 55 years especially the 25–34 years age group.

**Conclusions:**

The future projections of T2DM in Syria put it amongst countries with the highest levels of T2DM worldwide. It is estimated that by 2022 approximately a fifth of the Syrian population aged 25 years and older will have T2DM.

## Background

Type 2 diabetes mellitus (T2DM) is a worldwide public health problem affecting populations at all levels of socioeconomic development. Diabetes is characterized by chronic hyperglycemia, and is frequently accompanied by dyslipidemia, hypertension, and endothelial dysfunction. Diabetes therefore, is a major risk factor for cardiovascular vascular disease [1].

The prevalence of T2DM is increasing worldwide and has reached alarming levels in many countries around the world. It is estimated that 366 million people were affected by diabetes in 2011 globally, and the number is projected to increase to 552 million in 2030 [2]. Low- and middle-income countries face the greatest threat of diabetes, where between 2010 and 2030 a 70% increase in the number of adults with diabetes is projected to take place compared to 20% for developed countries [3]. This increase has been linked to population growth and aging, as well as increasing urbanization, obesity and physical inactivity [4,5].

Still, many governments and public health planners in developing countries remain largely unaware of the current magnitude and future burden of diabetes in their societies [2]. Projecting future trends in diabetes in developing counties, therefore, can help in guiding a proportionate public health response to the diabetes epidemic.

Syria is going through many of the changes that can influence diabetes trends in a negative way. Still, no study to date has attempted to use available national data to forecast future trends in diabetes in Syria. The paucity of data sources in Syria prompted the use and adaptation of the IMPACT Diabetes model that requires fewer data inputs to project T2DM prevalence among adults in Syria over the period 2003 to 2022, and to quantify the potential impact of public health interventions aimed to reduce two of its major risk factors; obesity and smoking.

## Methods

### The model

**Figure 1:**
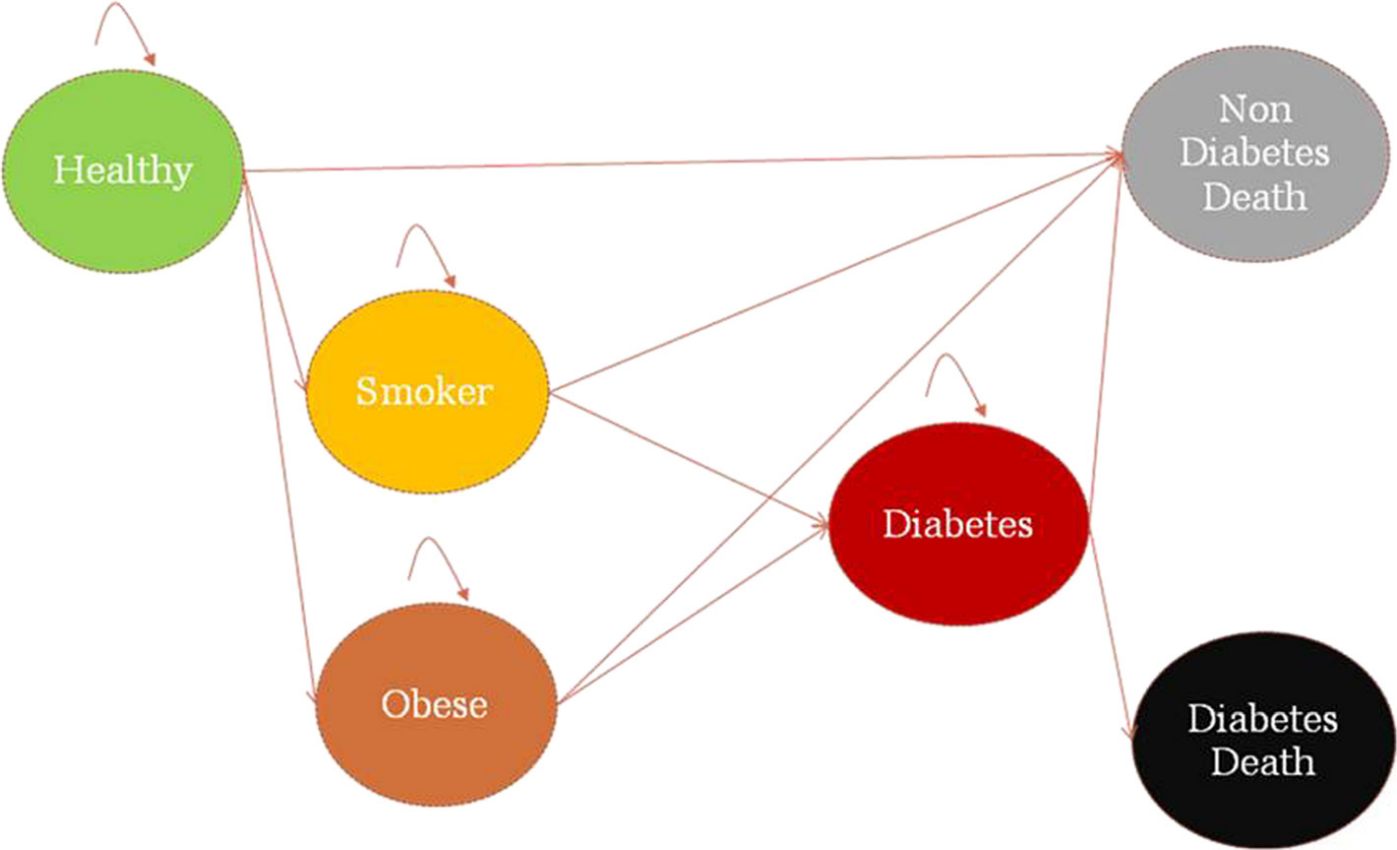
The MEDCHAMPS IMPACT Diabetes model structure.

Future diabetes prevalence in Syria was estimated using the IMPACT Diabetes Model that was developed for this purpose through the MedCHAMPS project [6,7]. Based on a Markov modelling approach [8], this model assumes that the population can be divided into several pools at an initial point of time defined as “starting states”: healthy people (e.g. non-obese, non-smokers, and have no diabetes), people who are obese, and people who are smokers. A proportion of the population moves from each starting state to the diabetes state after each cycle lasting 1 year and finally to two “absorbing states” that take into account competing risk for mortality (diabetes-related deaths and non-diabetes-related deaths) (Figure 1). Potential overlaps between healthy, obese and smoking groups have been taken into account by estimating conditional probabilities of group membership. Furthermore, the model assumes that there is no “remission” of people in the diabetes state towards the previous states.

Demographic and risk factors trends in the population are used to inform the relative size of the “starting states” and its change over time. Transition probabilities are used to estimate the proportion of persons moving from the starting states to the diabetes or death states. (Additional file 1: Table S4).

### Data sets and estimation of key model parameters

The population structure at the baseline year (2003) was obtained from the Syrian Bureau of Statistics [9]. For subsequent years, population trends were obtained from the United Nations Department of Economic and Social Affairs [10], which predicts an almost doubling of the Syrian population between 2003 and 2022. The increase during this period is expected to be smallest for the youngest age group (25–34 years), and highest for the age group of 55–64 years. The age group of 75 years and over is projected to have more women than men, although the overall number of men is projected to be slightly higher than women.

The prevalence of diabetes, obesity and smoking at the baseline year were obtained from the STEPwise survey (2003) in Syria [11]. In this nationwide survey involving 9184 participants, diabetes was defined as self-reported diabetes multiplied by 1.5 to account for undiagnosed cases [2]. Obesity was defined as having a body mass index (BMI) higher or equal to 30. Smoking was defined as current daily cigarette smoking. Obesity trends were roughly estimated by extrapolation from local unpublished data, whereby obesity prevalence was estimated to have increased by 1% per year for all age groups except for the 25–35 years age group, where the increase was estimated to be at 0.2% per year. The obesity increase was assumed to peak at 80% prevalence [12,13]. Smoking trends were determined based on experts’ interviews conducted by the MedCHAMPS project, in which a no-change scenario was used. The baseline prevalences of DM, obesity and smoking in 2003 were: 9.2% in men and 14.7% in women for diabetes, 29% in men and 40.1% in women for obesity, and 59.1% in men and 18.8% in women for smoking. Total mortality was obtained from the United Nations Department of economic and social affairs [10].

The population incidence of diabetes and diabetes-related excess mortality were estimated using DisMod II software [14,15], and were used to estimate key transition probabilities. DisMod II can provide estimates of case fatality and incidence for a disease if prevalence, remission rate, and total and disease specific mortality rates are known. For this study, we used diabetes prevalence in 2003, diabetes remission rate (which can be reasonably assumed to be 0), and relative risk (RR) of mortality from diabetes in 2003 for the Syrian population. The latter parameter was estimated as proposed by Barendregt et al. [16], based on diabetes prevalence in 2003 and usual RR for mortality (mortality in diseased/mortality in non-diseased) obtained from the Verona study [17]. The RR of diabetes in obese and smoking individuals were obtained from two recent systematic reviews and meta-analysis [18,19]. The data used for this study is openly available. More details on the data and on the calculation of the incidence parameter are available in the technical appendix (Additional file 1).

### Comparison with the projections of international diabetes federation (IDF)

The IDF estimated future diabetes prevalence among people 20–79 years of age, while age range in the model output for this study was 25 years and over. Therefore, to allow better comparison with IDF estimates, prevalence of diabetes was recalculated from the model outputs after excluding all people >79 years of age, and adding number of people 20–24 years of age for each year assuming an extremely low prevalence of T2DM in this age band.

### Estimating the potential gains of public health interventions to prevent T2DM

To assess the potential effect of a likely successful policy for diabetes prevention, the model was re-run after a scenario of a hypothetical 10-year intervention program (started in 2003) was included. The hypothetical prevention program was assumed to target a group of non-diabetic persons (10% of healthy, 40% of obese, and 20% of smokers) for an expected reduction of 25% of obesity and 50% of smoking in 10 years.

## Results

### Diabetes incidence, case fatality and mortality

According to the DisMod II outputs, the estimated annual diabetes incidence was higher in women than in men (1570 per 100,000 vs. 1190 per 100,000). The same was found for diabetes-related mortality rate (70 per 100,000 in women vs. 50 per 100,000 in men). The rates showed an increasing trend with age for both diabetes incidence (from 405 per 100,000 in the 25–34 year age group to 5085 per 100,000 in the ≥75 year age group), and diabetes mortality rate (from 0 to 1120 per 100,000 in the 25–34 year and the ≥75 year age groups, respectively). (Additional file 1: Table S4)

### Projected prevalence of diabetes

**Figure 2:**
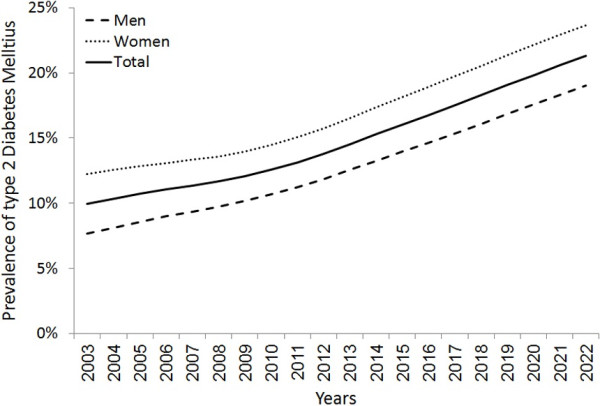
Projected trend of type 2 diabetes mellitus prevalence in Syria from 2003 to 2022, by gender.

**Figure 3:**
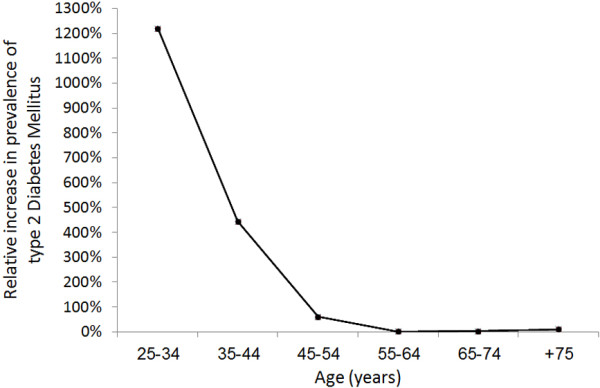
The relative increase in prevalence of type 2 diabetes mellitus between 2003 and 2022 among Syrian adults stratified by age.

Overall, the model predicts a relative increase of 113.8% in diabetes prevalence (from10% to 21.3%), and of 329.2% in number of people with diabetes (from 686,195 to 2,944,813) between 2003 and 2022. (Table 1 and Figure 2) In women, the prevalence of and number of patients with diabetes are estimated to be 12.2% (423,694) in 2003 and 23.6% (1,611,729) in 2022. In men, the numbers are lower; 7.7% (262,500) in 2003 and 19% (1,333,084) in 2022. However, the relative increase in the prevalence of diabetes and number of diabetic people is higher in men than in women (148.2% and 407.8% in men vs. 93.2% and 280.4% in women) during the same period. Most of the increase in the prevalence of diabetes is projected to occur in younger age groups, where diabetes is estimated to increase by 12.2 and 4.4 folds among the age groups of 25–34 years and 35–44 years, respectively, as opposed to almost zero increase in the age group of 55–64 years and above (Table 1 and Figure 3). Among people 20–79 years of age, and between 2011 and 2022, the model predicts a relative increase of 69.4% in diabetes prevalence (from 10.9% to 18.4%), and of 115% in number of people with diabetes (from 1,357,454 to 2,918,314) (Table 2).

**Table 1:** Projected prevalence and number of people with type 2 diabetes for selected years among adults 25 years of age and over in Syria

		**2003**	**2006**	**2011**	**2022**
**Men**	**25-34**	16995 (1.2)	37073 (2.2)	87962 (4)	249919 (11.3)
	**35-44**	28483 (3.2)	57424 (5.4)	119992 (8.6)	325066 (15.7)
	**45-54**	66424 (12.2)	91985 (14.3)	146472 (17.4)	329564 (23.9)
	**55-64**	82300 (27)	95620 (27.2)	126473 (26.7)	245785 (30.5)
	**65-74**	47502 (27)	56226 (28.8)	73564 (31.5)	129752 (33.3)
	**75+**	20797 (27)	24638 (28)	32497 (30.9)	52998 (37)
	**total**	262500 (7.7)	362966 (9)	586960 (11.2)	1333084 (19)
**Women**	**25-34**	6682 (0.5)	26976 (1.6)	77663 (3.7)	231184 (10.9)
	**35-44**	29348 (3.3)	63648 (6.1)	139289 (10.2)	388171 (19.8)
	**45-54**	125064 (22.4)	151993 (23.1)	212463 (25.3)	419033 (32.1)
	**55-64**	134372 (42.3)	148746 (40.7)	185811 (37.7)	322827 (40.7)
	**65-74**	87573 (42.3)	97922 (43.1)	117106 (44.6)	167225 (38.9)
	**75+**	40655 (42.3)	43758 (38.9)	54410 (37.9)	83289 (41)
	**total**	423694 (12.2)	533044 (13.1)	786742 (15.1)	1611729 (23.6)
**Total**	**25-34**	23678 (0.8)	64049 (1.9)	165624 (3.9)	481102 (11.1)
	**35-44**	57831 (3.2)	121072 (5.7)	259281 (9.4)	713236 (17.7)
	**45-54**	191488 (17.3)	243978 (18.7)	358935 (21.3)	748597 (27.9)
	**55-64**	216672 (34.8)	244366 (34)	312284 (32.3)	568612 (35.6)
	**65-74**	135075 (35.3)	154148 (36.5)	190670 (38.5)	296977 (36.2)
	**75+**	61452 (35.5)	68396 (34.1)	86908 (34.9)	136287 (39.3)
	**total**	686195 (10)	896010 (11)	1373702 (13.1)	2944813 (21.3)

Data is shown as number and prevalence (%).

**Table 2:** Projected prevalence and number of people with type 2 diabetes for selected years among adults 20–79 years of age in Syria

		**2003**	**2006**	**2011**	**2022**
**Men**	20-24*	0 (0)	0 (0)	0 (0)	0 (0)
	**25-34**	16995 (1.2)	37073 (2.2)	87962 (4)	249919 (11.3)
	**35-44**	28483 (3.2)	57424 (5.4)	119992 (8.6)	325066 (15.7)
	**45-54**	66424 (12.2)	91985 (14.3)	146472 (17.4)	329564 (23.9)
	**55-64**	82300 (27)	95620 (27.2)	126473 (26.7)	245785 (30.5)
	**65-74**	47502 (27)	56226 (28.8)	73564 (31.5)	129752 (33.3)
	**75-79**	10398 (27)	12319 (28)	16249 (30.9)	26499 (37)
	**total**	252102 (6.2)	350647 (7.2)	570712 (9.1)	1306585 (16.2)
**Women**	20-24*	0 (0)	0 (0)	0 (0)	0 (0)
	**25-34**	6682 (0.5)	26976 (1.6)	77663 (3.7)	231184 (10.9)
	**35-44**	29348 (3.3)	63648 (6.1)	139289 (10.2)	388171 (19.8)
	**45-54**	125064 (22.4)	151993 (23.1)	212463 (25.3)	419033 (32.1)
	**55-64**	134372 (42.3)	148746 (40.7)	185811 (37.7)	322827 (40.7)
	**65-74**	87573 (42.3)	97922 (43.1)	117106 (44.6)	167225 (38.9)
	**75-79**	40655 (84.6)	43758 (77.8)	54410 (75.8)	83289 (82)
	**total**	423694 (10.3)	533044 (11)	786742 (12.7)	1611729 (20.7)
**Total**	20-24*	0 (0)	0 (0)	0 (0)	0 (0)
	**25-34**	23678 (0.8)	64049 (1.9)	165624 (3.9)	481102 (11.1)
	**35-44**	57831 (3.2)	121072 (5.7)	259281 (9.4)	713236 (17.7)
	**45-54**	191488 (17.3)	243978 (18.7)	358935 (21.3)	748597 (27.9)
	**55-64**	216672 (34.8)	244366 (34)	312284 (32.3)	568612 (35.6)
	**65-74**	135075 (35.3)	154148 (36.5)	190670 (38.5)	296977 (36.2)
	**75-79**	51053 (58.9)	56077 (55.9)	70659 (56.8)	109788 (63.4)
	**total**	675796 (8.2)	883691 (9.1)	1357454 (10.9)	2918314 (18.4)

Data is shown as number and prevalence (%). * This young age group was assumed to have no cases of type 2 diabetes mellitus.

**Figure 4:**
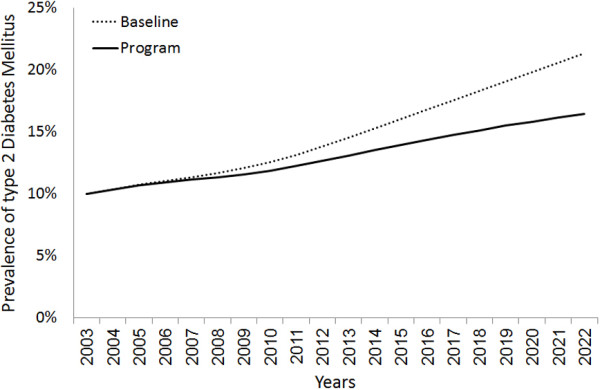
**Predicted effect of a hypothetical 10-years prevention program (starting in 2003) on prevalence of type 2 diabetes mellitus.** The hypothetical prevention program used here is assumed to target a group of non-diabetic persons (10% of healthy, 40% of obese, and 20% of smoker) with an expected reduction of 25% of obesity and 50% of smoking in 10 years.

If the scenario of 10-years prevention program described above is initiated, the model predicts a small but useful reduction in diabetes prevalence and diabetics after 5 years, from 11.3% (977,938) to 11.1% (975,881). More notable changes in diabetes prevalence and diabetics as a result of the intervention are predicted to occur later, with a reduction from 13.8% (1,488,379) to 12.6% (1,362,617) after 10 years, and from 21.3% (2,944,813) to 16.4% (2,267,905) after 20 years (Figure 4).

## Discussion

This is the first modelling study forecasting diabetes prevalence in Syria using primarily local data. Starting in 2003, our model predicted that the number of individuals with diabetes would increase four folds, and diabetes prevalence would double in about 20 years.

The IDF projections predicted a relative increase of 14.6% (from 8.2% to 9.4%) in diabetes prevalence, and 91.9% (from 889,500 to 1,706,700) in the number of diabetics among Syrian adults 20–79 years of age between 2011 and 2030 [2]. These estimates are substantially lower than ours. Only between 2011 and 2022, our model predicted a higher increase of 69.4% in diabetes prevalence and of 115% in number of diabetics (Table 2). These differences may be due to the absence of local data inputs in the IDF projections. The IDF prevalence estimates were therefore generated using an average of available data from countries matched by IDF region, World Bank’s income classification group, geography, and ethnicity [20]. Another reason for this discrepancy could be due to diabetes-related mortality estimates. Deaths attributed to diabetes in Syria were estimated by the IDF at 6,066 in 2011, while a lower estimate (4,841) of diabetes mortality for the same year was found in our study using the DisMod II model.

Our results suggest that the increase in diabetes prevalence and number of diabetics in Syria will be substantially higher than in many other developing countries in the Middle Eastern and North African regions [3]. Our projections are also higher than those reported for developed countries. For example, the IDF projected an increase in prevalence of diabetes from 10.9 in 2011 to 11.8% in 2030 in the United States, from 7.3% to 8.8% in the Netherlands, and from 6.8% to 7.5% in the United Kingdom [2].

Interestingly, our results predict that the increase in diabetes prevalence and number of individuals with diabetes will be higher in younger age groups (< 55 years), especially for women (Figure 3). This is consistent with the literature predicting an increase in diabetes risks in younger adults in non-western societies [21]. Therefore, a special emphasis in any efforts to reduce diabetes burden in the Syrian and similar societies should be directed towards these age groups.

Another interesting population trend in diabetes is related to the urban–rural distribution. For example, in developed countries the prevalence of diabetes is reported to be similar in rural and urban areas [22]. For most developing countries however, the prevalence of diabetes in rural areas is assumed to be one-half that of urban areas [23]. The model used in this study did not make assumptions and projections regarding diabetes prevalence in rural vs. urban regions. However, a recent study from Syria estimated the prevalence of diabetes among urban population ≥ 25 years of age (2006) to be at 15.6% [24]. In this report, the model-estimated prevalence of diabetes in the total population in 2006 is 11%. Assuming roughly equal distribution of the Syrian population between rural and urban areas [9], the prevalence of diabetes among rural populations in Syria is likely to be substantially lower than that of urban areas (approximately 6.5%). This suggests that the pattern of diabetes distribution in Syria is similar to that in other developing countries, and a prominent role for urban lifestyles in diabetes morbidity.

Our intervention modelling shows that an important impact on diabetes prevalence could be achieved if an intervention program was implemented in 2003 to reduce the levels of obesity (by 25%) and smoking (by 50%) within 10 years. Although such reductions in obesity and smoking are hard to achieve in practice, the resulting reduction of 23% in diabetes prevalence by 2022 (a reduction of more than half a million diabetes cases) provides policy makers with some reliable benchmarks to gauge the size and impact of any planned intervention to reduce diabetes in the Syrian society.

Clearly, both IDF and the current study have methodological limitations. Our diabetes prevalence forecasting was based on trends in obesity and smoking, but did not include any other risk factors such as physical activity. No data on obesity and smoking trends is available in Syria. Therefore, the obesity trends used in this study were assumed based on cross sectional data combined with a rough extrapolation of local unpublished data consistent with the global increasing trend in obesity levels [25]. On the other hand, smoking trends were assumed to be steady in each age group for both genders. Moreover, the data from the STEPwise survey in Syria (2003) were not stratified by urban and rural status, precluding us to model this important factor. Finally, no diabetes projections could be obtained for the 20–24 year age group because no data on diabetes for this age group exist for Syria. This has prevented us from conducting a more robust comparison with IDF projections, which included people 20–79 years of age. However, the inclusion of this age group in our model output with the assumption of very low prevalence of diabetes along with the exclusion of people > 79 years of age allowed for some valid comparisons to be made between our and IDF’s projections.

## Conclusions

This study provides the first future projections of diabetes in Syria based on national data and epidemiological modelling techniques. Our data show that the burden of diabetes in Syria will markedly increase in the future potentially putting Syria amongst the top ten countries in diabetes prevalence worldwide. It also shows that our projected increases in diabetes burden surpass those of the IDF, highlighting the importance of local data in informing future projections. Available interventions can thwart this bleak forecast, but they will require extensive and sustained efforts targeting obesity and smoking in the Syrian society.

## Competing interests

The authors declared that they have no competing interest.

## Authors’ contributions

RA researched data and wrote the manuscript. FM reviewed/edited the manuscript. SR researched data. FMF contributed to the discussion and reviewed the manuscript. MO developed the original model and supported RA, SR, FMF, and WM. SC contributed to discussion and reviewed/edited the manuscript. WM reviewed the manuscript and supervised the work. All authors read and approved the final manuscript.

## Pre-publication history

The pre-publication history for this paper can be accessed here:

http://www.biomedcentral.com/1471-2458/13/507/prepub

## Supplementary Material

Additional file 1 **The IMPACT diabetes Model for MEDCHAMPS COUNTRY TECHNICAL APPENDIX-Syria.** This document describes the structure of the MEDCHAMPS IMPACT Diabetes model and related assumptions used in this study for projecting the future prevalence of type 2 diabetes mellitus in Syria in addition to the model output results. Click here for file
