# Supplementary material for: Forecasting future prevalence of type 2 diabetes mellitus in Syria
Source: BMC Public Health. 2013 May 25;13:507. doi: 10.1186/1471-2458-13-507 (PMC3673829; doi:10.1186/1471-2458-13-507)
Supplement: Additional file 1 — The IMPACT diabetes Model for MEDCHAMPS COUNTRY TECHNICAL APPENDIX-Syria. This document describes the structure of the MEDCHAMPS IMPACT Diabetes model and related assumptions used in this study for projecting the future prevalence of type 2 diabetes mellitus in Syria in addition to the model output results. [file 1471-2458-13-507-S1.docx]

The IMPACT diabetes Model for SYRIA

Technical Appendix for the Baseline Model

Samer Rastam

Radwan Al Ali

Fouad M.Fouad

Wasim Maziak

Martin O’Flaherty

Simon Capewell

Julia Critchley

Nigel Unwin

On behalf of the MEDCHAMPS Project

July / 2011

Contents

[1. The Model 2](#_Toc351597073)

[Methods Overview 2](#_Toc351597074)

[The Model Structure: 2](#_Toc351597075)

[The Model Workbook 3](#_Toc351597076)

[Sensitivity Analysis: 4](#_Toc351597077)

[2. Data needs 4](#_Toc351597078)

[Minimum dataset 4](#_Toc351597079)

[Deriving model parameters 5](#_Toc351597080)

[3. Model Validation 8](#_Toc351597081)

[Background and aims: 8](#_Toc351597082)

[Results 8](#_Toc351597083)

[4. Data Sources and assumptions. 10](#_Toc351597084)

[5. Model inputs 11](#_Toc351597085)

[6. Model outputs 15](#_Toc351597086)

[References: 16](#_Toc351597087)

# The Model

The purpose of the MEDCHAMPS IMPACT Diabetes model is to provide estimates of future diabetes prevalence and offer a modelling platform for policy decision making.

## Methods Overview

The model integrates information on population, obesity and smoking trends at a given point in time to estimate diabetes prevalence in the future.

The population is partitioned in three states (healthy, obese and smokers) and from them, number of diabetes patients and diabetic and non diabetic deaths are estimated for subsequent time periods using a Markov approach.

The effect of policy decisions can be modelled by the estimated effect on risk factors trends, and the trend parameter can be modified to model increasing, decreasing or stable trends in the prevalence of obesity and or smoking. Another way of exploring policy options (like, for example, multifactorial lifestyle diabetes prevention interventions) can be modelled through the modifications of the diabetes incidence parameter.

## The Model Structure:

Models are simplifications of reality. In order to keep the model simple but at the same time useful, many compromises on the way the disease epidemiology is modelled are necessary. More complex models usually requires different approaches and an amount of data that probably will not be available for the participating countries. A graphical description of the model is presented in figure 1.

We assume that the population can be divided in several pools: Diabetes mellitus, Obese, Smoker and “healthy” (eg: non obese, non smokers, non diabetics). A proportion of the population in each pool moves through pathways to other states as described in figure 1. Population demographic trends are used to inform the relative size of the “starting states”, and transition probabilities are used to estimate the proportion of persons moving from the starting states to the diabetes and death states. There are two “absorbing states” : Diabetes Mellitus (DM)related death and Non DM related deaths. In this way, mortality competing risk are modelled. Potential overlaps between the healthy, obese and smoking group are managed by calculating the conditional probabilities of membership.

Figure 1. The MEDCHAMPS IMPACT Diabetes Model Structure.


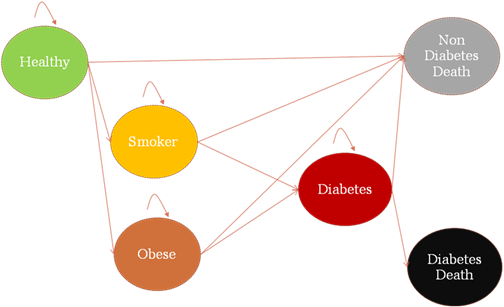


## The Model Workbook

The workbook is a MS Excel spreadsheet, structured in tabs. These tabs serve different purposes, but for the end-user the key tabs are the Data Input , Dashboard and Validation tabs.

The following is a more detailed description of each tab and its purpose, with a more thorough description of the key ones.

***The Data Input tab:***

The data to be imputed is highlighted in gray shaded cells. The following sections are available:

Population data: used to input the age and gender structure of the population being modelled, and the populations projections

Morbidity data: cross sectional data on diabetes prevalence, obesity and smoking is provided. Trend parameters can be set up here also or use the defaults. Currently, linear trends can be modelled, but any other type of trend can be implemented.

***The Dashboard tab:***Figures and tables presenting the model estimated diabetes prevalence are presented here.

***The validation tab:***This tab summarizes important validation information for the country, if the validation exercise has been conducted. .

***Outputs:***

The raw outputs of the model and the sensitivity analysis is stored here. Available information:numbers of diabetes patients , diabetes prevalence , minimun and maximun estimates.

***SA layer:***

Calculations for estimating transition probabilities are performed here. It also provides functionality to run the sensitivity analysis macro, by pressing CTRL+E.

***Markov Chains:***

They are implemented in separate tabs for each gender and age group. Two sets of chains are implemented here for the baseline and scenario runs.

## Sensitivity Analysis:

We used the analysis of the extremes method [[1](#_ENREF_1)], consisting in running the model with all parameters set to a minimum and maximum realistic values. This is a very conservative approach, but allows a more transparent understanding of the weight of each parameter regarding model outcomes. The Sensitivity Analysis is updated by running a macro (press CTRL+E).

# Data needs

## Minimum dataset

The model requires data by 10 year age and gender bands, starting at 25, ending 75+. Details on the sources are available in the table in section 4. The actual values used in the model are presented in section 5.

1. Initial year:
   1. Population
   2. Diabetes prevalence
   3. Obesity prevalence
   4. Smoking prevalence (current smokers)
   5. Total mortality (needed for DISMOD based estimation of incidence rate)
2. Subsequent years for validation purposes
   1. Population
   2. Diabetes prevalence (as many time points as possible, particularly the latest available year)
   3. Obesity prevalence (as many time points as possible)
   4. Smoking prevalence (as many time points as possible)
3. Subsequent year for forecasting purposes
   1. Population projections
   2. Obesity trends (assumption, initially a assuming a linear increase per year will be useful. We can also extrapolate from the existing trend data)
   3. Smoking trends (assumption, initially a assuming a linear increase per year will be useful. We can also extrapolate from the existing trend data)
4. DISMOD INPUTS:
   1. Incidence
   2. Case Fatality
   3. Mortality

Details of the data used are found in the data sources section (Section 4).

## Deriving model parameters

One of the key aims of the model is to use the minimum data requirements possible. We adapted the methods developed by Barendregt et [[2](#_ENREF_2)] to estimate two of the key parameters.

#### Diabetes incidence and specific mortality

The MEDCHAMPS Diabetes markov model use diabetes mellitus incidence and mortality as one of the critical data inputs that need to be provided by the participants countries, to help localize and calibrate the model to each population.

Since reliable and country specific sources of incidence data are probably not available, an estimate of it is needed.

We adapted a method to estimate it, and provide as an example, the estimation of baseline diabetes incidence for SYRIA

The method

Incidence, mortality and prevalence are closely related to each other, in a way that only some values for each parameter are consistent with the other parameters at a given time. This property has been used by Barendregt et al to estimate diabetes mellitus incidence in the Netherlands [[2](#_ENREF_2)].

The technique use as input whatever parameters that are known and using a multistate generic disease model using a lifetable markov approach, estimate revised parameters for the inputed ones and estimates for those unknown. This method has been implemented in a software called DISMOD II [[3](#_ENREF_3), [4](#_ENREF_4)].

For the MEDCHAMPS project, it is expected that the only available parameter is probably diabetes mellitus prevalence (either self reported or using ADA/NHANES definitions). However, diabetes excess mortality can be estimated form total mortality data (See Barendregt) using literature based estimates of mortality relative risk and disease prevalence, and we can safely assume that the remission rate for diabetes in effectively 0. Thus, the only parameters needed (by age and gender) are diabetes mellitus prevalence, population structure and population general mortality.

An important assumption is that this method requires a population in equilibrium, since the consistency between epidemiological estimates depends on the underlying trends in each parameter. However it is difficult to disentangle these effects from data inaccuracy. The robustness of the approach to violations of these assumptions is not known.

This method produces a “population incidence”, e.g., the incidence both for exposed and unexposed people to diabetes risk factors.

However, the MEDCHAMPS diabetes model needs incidence in the non-exposed, since incidence for obese persons and smokers is derived from that baseline incidence by using literature based relative risks.

It has been proposed that the incidence of a disease in a population is a weighted sum of the incidence among the exposed and the incidence among the unexposed to a risk factor [[5](#_ENREF_5)] (equation 1).

(Equation 1)

,


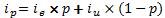

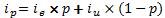

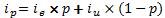

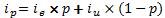

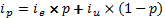

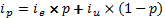


Where *i_p_* is the population incidence*, ie* is the incidence amongst the exposed*, i_u_* is the incidence amongst the unexposed and *p* is risk factor prevalence.

*Since the incidence in the exposed is the incidence in the unexposed times the relative risk (RR) (Equation 2),*

(equation 2)

,


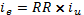

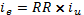


it is possible to derive from this two ideas the value for the unexposed incidence from the incidence in the population. Replacing equation 2 in equation 1

(Equation 3)


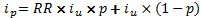


And then extracting I_u_ (Equation 4)

(Equation 4)


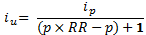


*Estimation of the incidence, case fatality and mortality parameters for SYRIA, 2003*

*Estimation of the population incidence:*

This section describes the method used to estimate diabetes mellitus type II incidence for the COUNTRY population in YEAR.

DISMOD needs at least 3 inputs. For this case, we used diabetes mellitus prevalence, diabetes mellitus remission rate and diabetes mellitus relative risk for mortality.

Diabetes and risk factors prevalence were obtained from three main studies:

1. Stepwise survey: This survey was conducted by Ministry of Health and World Health Organization in 2003. A national representative sample of 9184 participants was selected from all regions of Syria. This survey collected data about:

- Obesity defined as having BMI higher or equal to 30
- Diabetes defined based on self-reported disease, and to get the total prevalence estimation we multiply the numbers by 1.5 [[6](#_ENREF_6)].
- Smoking defined as current daily cigarette smoking.

Data were presented by gender and age groups with 10-years interval, starting from the age of 20 years. [[7](#_ENREF_7)]

1. Aleppo Household Survey (HEED): this is a cross-sectional survey conduced in 2004 by Syrian Centre for Tobacco Studies. The target population was adults 18-65 years residing in Aleppo. A multistage sampling was used with a total sample size of 2038. This survey collected data about:

- Obesity defined as having BMI higher or equal to 30
- Diabetes defined based on self-reported disease.
- Smoking defined as current daily cigarette smoking. [[8](#_ENREF_8)]

1. Aleppo diabetes survey: This survey was a cross-sectional survey conducted in 2006 by Syrian Centre for Tobacco Studies. The target population in this survey was adults age >=25 years residing in Aleppo. A Two-stage cluster sampling was used with a total sample size of 1168. This survey collected data about:

- Obesity defined as having BMI higher or equal to 30.
- Diabetes defined as a reported history of physician-diagnosed type 2 diabetes, or a fasting plasma glucose (FPG) levels >= 126 mg that was measured during the survey.
- Smoking defined as current daily cigarette smoking. [[9](#_ENREF_9)]

We can safely assume that diabetes mellitus remission rate is 0, and diabetes mellitus relative risk for mortality can be estimated as proposed by Barendregt et al [[2](#_ENREF_2)], based in the usual RR for mortality (mortality in diseased/mortality in non-diseased) and disease prevalence. The formula is

| (Equation 5) | $RR_{ADJ}=\frac{RR}{pRR+1-p}$ |
| --- | --- |

Where RR adj is the relative risk mortality, RR is the usual relative risk for mortality (mortality diseased/mortality healthy) and p is disease prevalence. The Verona Study [[10](#_ENREF_10)] provides age and gender specific values for RR. A summary of the calculations for this parameter is presented in table1.

Table 1. Estimating RRadj

| Age | Year | Verona RR | DM Prevalence | RRadj |
| --- | --- | --- | --- | --- |
|  |  |  | 2003 |  |
|  |  |  |  |  |
| 25-34 | men | 2.33 | 1.2% | 2.30 |
|  | women | 3.43 | 0.5% | 3.34 |
|  |  |  |  |  |
| 35-44 | men | 2.33 | 3.2% | 2.26 |
|  | women | 3.43 | 3.3% | 3.32 |
|  |  |  |  |  |
| 45-54 | men | 2.33 | 12.2 | 2.20 |
|  | women | 3.43 | 22.4 | 3.24 |
|  |  |  |  |  |
| 55-64 | men | 2.13 | 27% | 1.94 |
|  | women | 2.33 | 42.3% | 2.19 |
|  |  |  |  |  |
| 65-74 | men | 1.5 | 27% | 1.43 |
|  | women | 2.27 | 42.3% | 2.02 |
|  |  |  |  |  |
| 75+ | men | 1.13 | 27% | 1.11 |
|  | women | 1.32 | 42.3% | 1.28 |

# Model Validation

## Background and aims:

Model Validation is an important aspect of any modelling exercise, frequently overlooked.

We developed a model for SYRIA, over the period 2003 to 2022.

During that period, a single subsequent survey was conducted in 2006 (Aleppo diabetes survey) and we compared the model outputs with the observed prevalence estimates.

## Results

The observed prevalence of diabetes mellitus in SYRIA in 2003 was 7.7% in men and 12.2% in women. (STEPWise Syria, 2003, WHO), the estimated prevalence would increase to 19% in men and 23.6% in women by 2022. The estimated prevalence of obesity would increase from 29% in 2003 to 45.1% in 2022 in men and from 40.1% to 54.9% in women. During the same period the estimated smoking prevalence would increase in women from 18.8% to 52.6%, while decrease slightly in men from 59.1% to 57.3%. The modelled and observed estimates of diabetes prevalence are shown in Table 2 and Figure 2.

Table 2. A comparison of model and observed estimates for total diabetes prevalence SYRIA 2003-2022

|  | Observed (min-max) |  | Model (min-max) |
| --- | --- | --- | --- |
| 2006- Men  2006 – Women  2006 - Total | 12.1 – 19.9  12.9 – 13.7  13.7 – 17.7 |  | 7.3 - 10.6  10.6 - 15.5  9 - 13.1 |

Figure 2. A comparison of model and observed estimates for diabetes prevalence by gender, SYRIA 2006

The differences between the model and the observed data are due to the fact that observed data were collected from an urban area (city of Aleppo) from formal neighbourhood only.

# Data Sources and assumptions (Table 3)

| Data Item | Source | Comments |
| --- | --- | --- |
| **1.       Initial year:** | | |
| 1.1.     Population | Syrian bureau of Statistics ,2003^*1^ | Bureau provides population structure by 5-age groups and gender. |
| 1.2.    Diabetes prevalence | STEPWise ,2003 | A big national survey (9184 participants) |
| 1.3.    Obesity prevalence | STEPWise ,2003 |  |
| 1.4.   Smoking prevalence (current smokers) | STEPWise ,2003 |  |
| 1.5.    Total mortality (needed for DISMOD based estimation of incidence rate) | United Nations Department of Economic and Social Affaris^*2^ | Mortality data are grouped by 5 age groups and by gender |
| **2.      Subsequent years for validation and forecasting purposes** | | |
| 3.1.    Population trends | United Nations Department of Economic and Social Affairs | UN provided three different scenarios with low, medium and high fertility rates, we used data for medium fertility rate. |
| 3.2 Diabetes trend | Aleppo diabetes survey | A local survey with 1168 participants. |
| 3.3.   Obesity trends | Assumption based on extrapolation of the 1996-2006 data (linear projection) | We estimated 1% increase per year for all age groups expect for the younger age group (25-34 years where we used 0.2% increase trend. [[11](#_ENREF_11)] We stopped increasing when we reach 80% prevalence, as its implausible to sustain over time higher obesity prevalences. There is recent evidence from the US suggesting that prevalence trends might “cap” at a certain peak level. [[12](#_ENREF_12)] |
| 3.4.   Smoking trends | Assumption based on experts interviews. | No – change scenario |

*1: Syrian Bureau of Statistics [[13](#_ENREF_13)]

*2: United Nations Department of Economic and Social Affairs [[14](#_ENREF_14)]

# Model inputs (Table 4)

| **SECTION 1: POPULATION DATA** | | | | | | | | | | | | | |
| --- | --- | --- | --- | --- | --- | --- | --- | --- | --- | --- | --- | --- | --- |
|  |  |  |  |  |  |  |  |  |  |  |  |  |  |
| Start year | 2003 |  |  |  |  |  |  |  |  |  |  |  |  |
| Data source: ONS | |  |  |  |  |  |  |  |  |  |  |  |  |
|  | Men |  |  |  |  |  |  | Women |  |  |  |  |  |
| **year** | **25-34** | **35-44** | **45-54** | **55-64** | **65-74** | **75+** |  | **25-34** | **35-44** | **45-54** | **55-64** | **65-74** | **75+** |
| **2003** | 1427589 | 892466 | 545016 | 305195 | 176152 | 77122 |  | 1394405 | 886970 | 559314 | 317606 | 206991 | 96093 |
| **2004** | 1508695 | 942102 | 576180 | 317928 | 181937 | 80700 |  | 1472993 | 934105 | 591038 | 329999 | 213450 | 100996 |
| **2005** | 1599979 | 997477 | 609554 | 333478 | 188285 | 84333 |  | 1562045 | 987173 | 624347 | 345863 | 220152 | 106451 |
| **2006** | 1702771 | 1059190 | 645300 | 352157 | 195370 | 88042 |  | 1663079 | 1046936 | 659350 | 365595 | 227269 | 112547 |
| **2007** | 1814313 | 1126508 | 683211 | 373688 | 203175 | 91785 |  | 1773208 | 1112524 | 695810 | 388792 | 234781 | 119170 |
| **2008** | 1926362 | 1196958 | 722597 | 397374 | 211311 | 95461 |  | 1883600 | 1180867 | 732966 | 414406 | 242394 | 125975 |
| **2009** | 2027874 | 1267156 | 762523 | 422192 | 219238 | 98941 |  | 1982415 | 1247776 | 769816 | 440929 | 249691 | 132500 |
| **2010** | 2110879 | 1334667 | 802358 | 447391 | 226630 | 102144 |  | 2061175 | 1310273 | 805699 | 467238 | 256452 | 138416 |
| **2011** | 2172547 | 1398200 | 841864 | 472805 | 233325 | 105061 |  | 2116799 | 1366928 | 840389 | 493112 | 262542 | 143645 |
| **2012** | 2214899 | 1458327 | 881346 | 498632 | 239633 | 107755 |  | 2151663 | 1418613 | 874257 | 518793 | 268298 | 148318 |
| **2013** | 2240924 | 1516728 | 921382 | 524924 | 246273 | 110312 |  | 2169602 | 1467474 | 907994 | 544193 | 274559 | 152599 |
| **2014** | 2255748 | 1576025 | 962825 | 551837 | 254241 | 112852 |  | 2176929 | 1516806 | 942604 | 569329 | 282450 | 156737 |
| **2015** | 2263515 | 1637945 | 1006339 | 579524 | 264273 | 115482 |  | 2178661 | 1568904 | 978905 | 594295 | 292798 | 160958 |
| **2016** | 2265045 | 1702616 | 1052006 | 607955 | 276595 | 118272 |  | 2175837 | 1623971 | 1016994 | 618941 | 305858 | 165348 |
| **2017** | 2260192 | 1768765 | 1099752 | 637173 | 291060 | 121289 |  | 2168101 | 1680808 | 1056903 | 643426 | 321444 | 169973 |
| **2018** | 2251199 | 1834997 | 1149901 | 667504 | 307554 | 124635 |  | 2157595 | 1738587 | 1099300 | 668565 | 339378 | 175024 |
| **2019** | 2240603 | 1899350 | 1202805 | 699377 | 325824 | 128429 |  | 2146612 | 1796028 | 1144966 | 695451 | 359307 | 180719 |
| **2020** | 2230600 | 1960163 | 1258661 | 733122 | 345655 | 132786 |  | 2137114 | 1851933 | 1194407 | 724892 | 380918 | 187254 |
| **2021** | 2222289 | 2017453 | 1317264 | 768904 | 367113 | 137750 |  | 2130250 | 1906565 | 1247620 | 757237 | 404294 | 194691 |
| **2022** | 2216645 | 2070958 | 1378353 | 806723 | 390212 | 143410 |  | 2126931 | 1959509 | 1304282 | 792360 | 429409 | 203133 |
|  |  |  |  |  |  |  |  |  |  |  |  |  |  |

| **SECTION 2: MORBIDITY DATA** | | | | | | | | | | | | | | | | | | | | | | | | | | |
| --- | --- | --- | --- | --- | --- | --- | --- | --- | --- | --- | --- | --- | --- | --- | --- | --- | --- | --- | --- | --- | --- | --- | --- | --- | --- | --- |
|  | |  |  | | |  | |  | |  | |  | |  | |  |  | |  | |  | |  | |  | |
| **Diabetes prevalence** | | | | | | | | | | | | | | | | | | | | | | | | | | |
| Correction factor for DM prevalence | | | | | | | |  | |  | |  | |  | |  |  | |  | |  | |  | |  | |
|  | **men** | | | 1.5 |  | |  | |  | |  | |  | | **women** | | | 1.5 | |  | |  | |  | |  |
|  | |  |  | | |  | |  | |  | |  | |  | |  |  | |  | |  | |  | |  | |
|  | | **25-34** | **35-44** | | | **45-54** | | **55-64** | | **65-74** | | **75+** | |  | | **25-34** | **35-44** | | **45-54** | | **55-64** | | **65-74** | | **75+** | |
| Source | | 0.008 | 0.021 | | | 0.081 | | 0.180 | | 0.180 | | 0.180 | |  | | 0.003 | 0.022 | | 0.149 | | 0.282 | | 0.282 | | 0.282 | |
|  | | HSE*1.5 (self reported adjustment) | | | | | | | |  | |  | |  | |  |  | |  | |  | |  | |  | |
| **Obesity prevalence trends (BMI >30)** | | | | | | | | | | | | | | | | | | | | | | | | | | |
| Data source: HSE | | |  | | |  | |  | |  | |  | |  | |  |  | |  | |  | |  | |  | |
| **year** | | Men |  | | |  | |  | |  | |  | |  | | Women |  | |  | |  | |  | |  | |
| **2003** | | 0.190 | 0.298 | | | 0.388 | | 0.438 | | 0.463 | | 0.407 | |  | | 0.210 | 0.354 | | 0.578 | | 0.728 | | 0.751 | | 0.722 | |
| **2004** | | 0.192 | 0.308 | | | 0.398 | | 0.448 | | 0.473 | | 0.417 | |  | | 0.212 | 0.364 | | 0.588 | | 0.738 | | 0.761 | | 0.732 | |
| **2005** | | 0.194 | 0.318 | | | 0.408 | | 0.458 | | 0.483 | | 0.427 | |  | | 0.214 | 0.374 | | 0.598 | | 0.748 | | 0.771 | | 0.742 | |
| **2006** | | 0.196 | 0.328 | | | 0.418 | | 0.468 | | 0.493 | | 0.437 | |  | | 0.216 | 0.384 | | 0.608 | | 0.758 | | 0.781 | | 0.752 | |
| **2007** | | 0.198 | 0.338 | | | 0.428 | | 0.478 | | 0.503 | | 0.447 | |  | | 0.218 | 0.394 | | 0.618 | | 0.768 | | 0.791 | | 0.762 | |
| **2008** | | 0.200 | 0.348 | | | 0.438 | | 0.488 | | 0.513 | | 0.457 | |  | | 0.220 | 0.404 | | 0.628 | | 0.778 | | 0.801 | | 0.772 | |
| **2009** | | 0.202 | 0.358 | | | 0.448 | | 0.498 | | 0.523 | | 0.467 | |  | | 0.222 | 0.414 | | 0.638 | | 0.788 | | 0.801 | | 0.782 | |
| **2010** | | 0.204 | 0.368 | | | 0.458 | | 0.508 | | 0.533 | | 0.477 | |  | | 0.224 | 0.424 | | 0.648 | | 0.798 | | 0.801 | | 0.792 | |
| **2011** | | 0.206 | 0.378 | | | 0.468 | | 0.518 | | 0.543 | | 0.487 | |  | | 0.226 | 0.434 | | 0.658 | | 0.808 | | 0.801 | | 0.802 | |
| **2012** | | 0.208 | 0.388 | | | 0.478 | | 0.528 | | 0.553 | | 0.497 | |  | | 0.228 | 0.444 | | 0.668 | | 0.808 | | 0.801 | | 0.802 | |
| **2013** | | 0.210 | 0.398 | | | 0.488 | | 0.538 | | 0.563 | | 0.507 | |  | | 0.230 | 0.454 | | 0.678 | | 0.808 | | 0.801 | | 0.802 | |
| **2014** | | 0.212 | 0.408 | | | 0.498 | | 0.548 | | 0.573 | | 0.517 | |  | | 0.232 | 0.464 | | 0.688 | | 0.808 | | 0.801 | | 0.802 | |
| **2015** | | 0.214 | 0.418 | | | 0.508 | | 0.558 | | 0.583 | | 0.527 | |  | | 0.234 | 0.474 | | 0.698 | | 0.808 | | 0.801 | | 0.802 | |
| **2016** | | 0.216 | 0.428 | | | 0.518 | | 0.568 | | 0.593 | | 0.537 | |  | | 0.236 | 0.484 | | 0.708 | | 0.808 | | 0.801 | | 0.802 | |
| **2017** | | 0.218 | 0.438 | | | 0.528 | | 0.578 | | 0.603 | | 0.547 | |  | | 0.238 | 0.494 | | 0.718 | | 0.808 | | 0.801 | | 0.802 | |
| **2018** | | 0.220 | 0.448 | | | 0.538 | | 0.588 | | 0.613 | | 0.557 | |  | | 0.240 | 0.504 | | 0.728 | | 0.808 | | 0.801 | | 0.802 | |
| **2019** | | 0.222 | 0.458 | | | 0.548 | | 0.598 | | 0.623 | | 0.567 | |  | | 0.242 | 0.514 | | 0.738 | | 0.808 | | 0.801 | | 0.802 | |
| **2020** | | 0.224 | 0.468 | | | 0.558 | | 0.608 | | 0.633 | | 0.577 | |  | | 0.244 | 0.524 | | 0.748 | | 0.808 | | 0.801 | | 0.802 | |
| **2021** | | 0.226 | 0.478 | | | 0.568 | | 0.618 | | 0.643 | | 0.587 | |  | | 0.246 | 0.534 | | 0.758 | | 0.808 | | 0.801 | | 0.802 | |
| **2022** | | 0.228 | 0.488 | | | 0.578 | | 0.628 | | 0.653 | | 0.597 | |  | | 0.248 | 0.544 | | 0.768 | | 0.808 | | 0.801 | | 0.802 | |
|  | |  |  | | |  | |  | |  | |  | |  | |  |  | |  | |  | |  | |  | |

| **Smoking Prevalence Trends** | | | | | | | | | | | | | |
| --- | --- | --- | --- | --- | --- | --- | --- | --- | --- | --- | --- | --- | --- |
|  | Men |  |  |  |  |  |  | Women |  |  |  |  |  |
| **year** | **25-34** | **35-44** | **45-54** | **55-64** | **65-74** | **75+** |  | **25-34** | **35-44** | **45-54** | **55-64** | **65-74** | **75+** |
| **2003** | 0.692 | 0.629 | 0.507 | 0.396 | 0.270 | 0.400 |  | 0.159 | 0.261 | 0.211 | 0.138 | 0.122 | 0.100 |
| **2004** | 0.692 | 0.629 | 0.507 | 0.396 | 0.270 | 0.400 |  | 0.159 | 0.261 | 0.211 | 0.138 | 0.122 | 0.100 |
| **2005** | 0.692 | 0.629 | 0.507 | 0.396 | 0.270 | 0.400 |  | 0.159 | 0.261 | 0.211 | 0.138 | 0.122 | 0.100 |
| **2006** | 0.692 | 0.629 | 0.507 | 0.396 | 0.270 | 0.400 |  | 0.159 | 0.261 | 0.211 | 0.138 | 0.122 | 0.100 |
| **2007** | 0.692 | 0.629 | 0.507 | 0.396 | 0.270 | 0.400 |  | 0.159 | 0.261 | 0.211 | 0.138 | 0.122 | 0.100 |
| **2008** | 0.692 | 0.629 | 0.507 | 0.396 | 0.270 | 0.400 |  | 0.159 | 0.261 | 0.211 | 0.138 | 0.122 | 0.100 |
| **2009** | 0.692 | 0.629 | 0.507 | 0.396 | 0.270 | 0.400 |  | 0.159 | 0.261 | 0.211 | 0.138 | 0.122 | 0.100 |
| **2010** | 0.692 | 0.629 | 0.507 | 0.396 | 0.270 | 0.400 |  | 0.159 | 0.261 | 0.211 | 0.138 | 0.122 | 0.100 |
| **2011** | 0.692 | 0.629 | 0.507 | 0.396 | 0.270 | 0.400 |  | 0.159 | 0.261 | 0.211 | 0.138 | 0.122 | 0.100 |
| **2012** | 0.692 | 0.629 | 0.507 | 0.396 | 0.270 | 0.400 |  | 0.159 | 0.261 | 0.211 | 0.138 | 0.122 | 0.100 |
| **2013** | 0.692 | 0.629 | 0.507 | 0.396 | 0.270 | 0.400 |  | 0.159 | 0.261 | 0.211 | 0.138 | 0.122 | 0.100 |
| **2014** | 0.692 | 0.629 | 0.507 | 0.396 | 0.270 | 0.400 |  | 0.159 | 0.261 | 0.211 | 0.138 | 0.122 | 0.100 |
| **2015** | 0.692 | 0.629 | 0.507 | 0.396 | 0.270 | 0.400 |  | 0.159 | 0.261 | 0.211 | 0.138 | 0.122 | 0.100 |
| **2016** | 0.692 | 0.629 | 0.507 | 0.396 | 0.270 | 0.400 |  | 0.159 | 0.261 | 0.211 | 0.138 | 0.122 | 0.100 |
| **2017** | 0.692 | 0.629 | 0.507 | 0.396 | 0.270 | 0.400 |  | 0.159 | 0.261 | 0.211 | 0.010 | 0.122 | 0.100 |
| **2018** | 0.692 | 0.629 | 0.507 | 0.396 | 0.270 | 0.400 |  | 0.159 | 0.261 | 0.211 | 0.010 | 0.122 | 0.100 |
| **2019** | 0.692 | 0.629 | 0.507 | 0.396 | 0.270 | 0.400 |  | 0.159 | 0.261 | 0.211 | 0.010 | 0.122 | 0.100 |
| **2020** | 0.692 | 0.629 | 0.507 | 0.396 | 0.270 | 0.400 |  | 0.159 | 0.261 | 0.211 | 1.010 | 0.122 | 0.100 |
| **2021** | 0.692 | 0.629 | 0.507 | 0.396 | 0.270 | 0.400 |  | 0.159 | 0.261 | 0.211 | 2.010 | 0.122 | 0.100 |
| **2022** | 0.692 | 0.629 | 0.507 | 0.396 | 0.270 | 0.400 |  | 0.159 | 0.261 | 0.211 | 3.010 | 0.122 | 0.100 |

Transition probabilities for incidence, diabetes specific mortality and total mortality (DisMod Calculations).

| Men |  |  |  |
| --- | --- | --- | --- |
|  | Incidence | Case Fatality | Mortality |
| 25-34 | 0.0038 | 0.0016 | 0 |
| 35-44 | 0.0101 | 0.0023 | 0.0001 |
| 45-54 | 0.0179 | 0.0039 | 0.0004 |
| 55-64 | 0.0248 | 0.0065 | 0.001 |
| 65-74 | 0.0316 | 0.0124 | 0.0027 |
| 75+ | 0.0425 | 0.0314 | 0.009 |
|  |  |  |  |
| All ages | 0.0119 | 0.0038 | 0.0005 |
| Women |  |  |  |
|  | Incidence | Case Fatality | Mortality |
| 25-34 | 0.0043 | 0.001 | 0 |
| 35-44 | 0.0128 | 0.0017 | 0.0001 |
| 45-54 | 0.0226 | 0.0026 | 0.0003 |
| 55-64 | 0.0317 | 0.0035 | 0.0007 |
| 65-74 | 0.0415 | 0.0086 | 0.0024 |
| 75+ | 0.0592 | 0.0368 | 0.0134 |
|  |  |  |  |
| All ages | 0.0157 | 0.0031 | 0.0007 |

# Model outputs

# References:

1. Briggs A, Sculpher M, Buxton M: **Uncertainty in the economic evaluation of health care technologies: the role of sensitivity analysis**. *Health Econ* 1994, **3**(2):95-104.

2. Barendregt JJ, Baan CA, Bonneux L: **An indirect estimate of the incidence of non-insulin-dependent diabetes mellitus**. *Epidemiology* 2000, **11**(3):274-279.

3. Barendregt JJ, Van Oortmarssen GJ, Vos T, Murray CJ: **A generic model for the assessment of disease epidemiology: the computational basis of DisMod II**. *Population health metrics* 2003, **1**(1):4.

4. World Health Organization: **Health statistics and health information systems. Global Burden of Disease (GBD)**. 2012(Avalable at <http://www.who.int/healthinfo/global_burden_disease/tools_software/en/)>.

5. Szklo M, Nieto FJ: **Epidemiology : beyond the basics**. Gaithersburg, Md: Aspen; 2000.

6. International Diabetes Federation: **IDF Diabetes Atlas. Fifth Edition.** 2011.

7. World Health Organization: **Noncommunicable diseases. STEPwise surveillance. Syrian Arab Republic Report. [**[**http://www.emro.who.int/ncd/pdf/stepwise_syria.pdf]**](http://www.emro.who.int/ncd/pdf/stepwise_syria.pdf%5d). 2003.

8. Maziak W, Ward KD, Mzayek F, Rastam S, Bachir ME, Fouad MF, Hammal F, Asfar T, Mock J, Nuwayhid I *et al*: **Mapping the health and environmental situation in informal zones in Aleppo, Syria: report from the Aleppo household survey**. *Int Arch Occup Environ Health* 2005, **78**(7):547-558.

9. Albache N, Al Ali R, Rastam S, Fouad FM, Mzayek F, Maziak W: **Epidemiology of Type 2 diabetes mellitus in Aleppo, Syria**. *J Diabetes* 2010, **2**(2):85-91.

10. Muggeo M, Verlato G, Bonora E, Bressan F, Girotto S, Corbellini M, Gemma ML, Moghetti P, Zenere M, Cacciatori V *et al*: **The Verona diabetes study: a population-based survey on known diabetes mellitus prevalence and 5-year all-cause mortality**. *Diabetologia* 1995, **38**(3):318-325.

11. Stevens GA, Singh GM, Lu Y, Danaei G, Lin JK, Finucane MM, Bahalim AN, McIntire RK, Gutierrez HR, Cowan M *et al*: **National, regional, and global trends in adult overweight and obesity prevalences**. *Population health metrics* 2012, **10**(1):22.

12. Schneider H, Dietrich ES, Venetz WP: **Trends and stabilization up to 2022 in overweight and obesity in Switzerland, comparison to France, UK, US and Australia**. *Int J Environ Res Public Health* 2010, **7**(2):460-472.

13. Central Bureau of Statistics: **Central Bureau of Statistics. [**[**http://www.cbssyr.org/index-EN.htm]**](http://www.cbssyr.org/index-EN.htm%5d). 2011.

14. United Nations: **Department of economics and social affairs. [**[**http://www.un.org/esa/population/**](http://www.un.org/esa/population/)**] . Accessed on 27 January 2011**. 2011.
